# Antibody–drug conjugates in cancer therapy: do we need biomarkers? A comprehensive review

**DOI:** 10.1007/s10555-026-10344-z

**Published:** 2026-05-26

**Authors:** Lei Lei, Ya-Bing Zheng, Shou-Ching Tang

**Affiliations:** 1 Department of Breast Medical Oncology, Zhejiang Cancer Hospital, No. 1 Banshan East Road, Hangzhou, 310022 People’s Republic of China; 2 LSU-LCMC Cancer Center, School of Medicine, Louisiana State University (LSU) Health Sciences Center, New Orleans, LA 70112 USA

**Keywords:** Antibody–drug conjugates, Linker cleavability, Biomarker dependence, Systemic toxicity, Tumor microenvironment, Bystander effect

## Abstract

Antibody–drug conjugates (ADCs) have revolutionized targeted cancer therapy, yet a fundamental clinical contradiction remains: while some ADCs require strict companion diagnostic testing, others demonstrate robust efficacy in biomarker-negative tumors. This review investigates the underlying mechanisms of this discrepancy, proposing that ADC linker cleavability is the primary determinant of both biomarker dependence and systemic toxicity profiles. By analyzing data from 40 clinical trials encompassing 7879 patients, we observed a distinct dichotomy based on linker design. ADCs utilizing noncleavable linkers function in a strictly biomarker-dependent manner, correlating with a targeted safety profile (34% severe toxicity rate) but relying entirely on antigen-mediated internalization. Conversely, ADCs with cleavable linkers facilitate premature payload release in the systemic circulation and tumor microenvironment. This enables a potent, biomarker-independent bystander effect—achieving a 29.7% tumor response rate even without target expression—but at the cost of significantly increased off-target systemic toxicities (47% severe toxicity rate). Recognizing this inherent trade-off between antitumor potency and safety, we further explore the development of next-generation, conditionally cleavable linkers. These advanced platforms are engineered for selective activation exclusively within the tumor microenvironment. We conclude that transitioning towards conditionally released linkers is essential to balance efficacy and systemic toxicity, optimizing the therapeutic index and fully realizing the potential of the “magic bullet.”

## Introduction

The foundational concept of antibody–drug conjugates (ADCs) dates back to Paul Ehrlich’s visionary “magic bullet” hypothesis, which was proposed over a century ago [1]. Modern ADCs realize this vision through three critical components: a tumor-targeting monoclonal antibody, a potent cytotoxic payload, and a precisely engineered linker. The advantage of ADCs includes the targeted delivery of cytotoxic drugs to tumors that bear specific membrane biomarkers as tumor antigens. Payloads are physically linked to monoclonal antibodies by linkers but remain inactive and nontoxic until they are released inside the biomarker-targeted tumor cells. Therefore, the systemic toxicities of ADCs should theoretically be negligible or at least lower than traditional cytotoxic drugs by targeted delivery of cytotoxic agents to biomarker-targeted tumor cells while sparing the surrounding biomarker-negative cells of normal tissues. Many ADCs have now been developed in oncology. They have demonstrated impressive efficacy against different malignancies from liquid to solid tumors. However, none of them were free of systemic toxicities [2, 3]. While some ADCs exhibited mild systemic toxicities [2], others were shown to have more toxicities. Some ADC payloads were shown to have systemic toxicities more severe than that of the parent prodrugs [4–6].

The exact mechanism of systemic toxicities from ADCs is currently unknown. Emerging data now demonstrate that the ADC design, especially the linker design may play an important role in the systemic toxicities from the ADCs [7] and biomarker dependence. We previously demonstrated that ADC with noncleavable linker likely releases the free payload from the lysed tumor cells, and the degree of their systemic toxicities was positively correlated with the treatment efficacy and patient survival [8]. Emerging literature from preclinical and clinical research now shows that ADCs with cleavable linkers produce more free payload in the blood and possess more systemic toxicities than those with non-cleavable linkers [8–11]. Importantly, recent preclinical data has shown that trastuzumab deruxtecan containing cleavable linker released the payload in the tumor microenvironment by cathepsin protease before reaching the tumor targets and resulted tumor destruction in a human epidermal growth factor receptor 2 (HER2)-independent manner [12]. Taken together, these observations strongly suggest that premature cleavage of payload from ADCs leads to more systemic toxicities and perhaps more bystander tumor destruction.

There is ongoing debate on the biomarker dependence or independence by which ADCs exert their antitumor effects. We specifically investigated whether target antigens must serve as necessary predictive biomarkers for patient selection and clinical response. Whereas some ADCs were approved for clinical application with a companion diagnostic biomarker testing [13, 14], many more ADCs were approved without the requirement for biomarker detection [15–18]. While some ADCs such as trastuzumab emtansine and transtuzumab deruxtecan target oncogene product HER2, others such as sacituzumab govitecan and mervetuximab soravtansine target housekeeping or nononcogenic products such as TROP-2 and folate receptor alpha (FRα), respectively. The housekeeping gene products are not exclusively tumor-specific; they also exhibit varied baseline expression in normal, healthy epithelial tissues. This differential antigen distribution between normal and malignant tissues profoundly impacts the therapeutic index, as potent payloads released via bystander effects or premature linker cleavage can lead to significant off-target toxicities among the healthy tissues that express the target proteins [19]. This review traces back the initial development and current commercially available ADCs, focusing on the ADC design, systemic toxicities, and the biomarker requirement. Our review links the cleavability of the ADC linker to its systemic toxicities and biomarker dependence and helps to design the next generation of ADCs to balance the efficacy and systemic toxicities.

## ADCs and systemic toxicities

The first clinical ADC prototype emerged in the 1980 s using an anti-carcinoembryonic antigen (CEA) murine antibodies linked to vindesine, but failed due to immunogenicity, linker instability, and excessive toxicity [20]. These early setbacks underscored the need for humanized antibodies and optimized linker chemistry, ultimately leading to the 2000 approval of gemtuzumab ozogamicin in 2000 for acute myeloid leukemia [21], though its subsequent withdrawal highlighted persistent challenges in therapeutic index optimization [22]. Following the successful approval of brentuximab vedotin in 2011 for hematologic malignancies, trastuzumab emtansine was the first modern ADC successfully developed in 2013 to treat HER2-positive metastatic breast cancer (mBC) [23]. Table 1 summarizes the key characteristics of FDA-approved ADCs to date.

**Table 1:** FDA-approved antibody–drug conjugates

Generic (trade) name	Target antigen	Linker	Payload	Initial FDA indication	FDA approval date	Pivotal clinical trial	Reference
Datopotamab deruxtecan (Datroway)	TROP2	Cleavable (GGFG, enzyme-responsive)	Deruxtecan (DXd)/TOP1 Inhibitor	HR +/HER2− metastatic breast cancer	Jan 2025	TROPION-Breast01	Bardia et al. [73]
Telisotuzumab vedotin (Emrelis)	c-Met	Cleavable (Val-Cit, Enzyme-responsive)	MMAE/microtubule inhibitor	c-Met-overexpressing NSCLC	Apr 2025	LUMINOSITY	Goldman et al. [74]
Mirvetuximab soravtansine (Elahere)	Folate receptor α (FRα)	Cleavable (disulfide)	DM4/microtubule inhibitor	Platinum-resistant ovarian cancer	Nov 2022	SORAYA	Matulonis et al. [75]
Tisotumab Vedotin (Tivdak)	Tissue factor (TF)	Cleavable (Val-Cit, enzyme-responsive)	MMAE/microtubule inhibitor	Recurrent/metastatic cervical cancer	Sep 2021	innovaTV 301	Vergote et al. [76]
Loncastuximab tesirine (Zynlonta)	CD19	Cleavable (Val-Ala, enzyme-responsive)	PBD dimer (SG3199)/DNA Crosslinker	Relapsed/refractory DLBCL	Apr 2021	LOTIS-2	Caimi et al. [77]
Sacituzumab govitecan (Trodelvy)	TROP2	Cleavable (CL2A, pH-sensitive)	SN-38/TOP1 inhibitor	Metastatic triple-negative breast cancer (mTNBC)	Apr 2020	ASCENT	Bardia et al. [39]
Trastuzumab deruxtecan (Enhertu)	HER2	Cleavable (GGFG, enzyme-responsive)	Deruxtecan (DXd)/TOP1 inhibitor	HER2 + unresectable/metastatic breast cancer	Dec 2019	DESTINY-Breast01	Modi et al. [23]
Enfortumab vedotin (Padcev)	Nectin-4	Cleavable (Val-Cit, enzyme-responsive)	MMAE/microtubule inhibitor	Locally advanced/metastatic urothelial carcinoma	Dec 2019	EV-201	Rosenberg et al. [78]
Polatuzumab vedotin (Polivy)	CD79b	Cleavable (Val-Cit, enzyme-responsive)	MMAE/microtubule inhibitor	Relapsed/refractory DLBCL (with BR)	Jun 2019	GO29365	Sehn et al. [79]
Moxetumomab pasudotox (Lumoxiti)	CD22	Cleavable	PE38 (Pseudomonas Exotoxin A)/Protein Inhibitor	Relapsed/refractory hairy cell leukemia (HCL)	Sep 2018	Study 1053	Kreitman et al. [80]
Inotuzumab ozogamicin (Besponsa)	CD22	Cleavable (acid-labile)	Calicheamicin/DNA cleavage agent	Relapsed/refractory B-cell ALL	Aug 2017	INO-VATE ALL	Kantarjian et al. [81]
Trastuzumab emtansine (Kadcyla)	HER2	Noncleavable (Thioether)	DM1/microtubule inhibitor	HER2 + metastatic breast cancer	Feb 2013	EMILIA	Verma et al. [82]
Brentuximab vedotin (Adcetris)	CD30	Cleavable (Val-Cit, enzyme-responsive)	MMAE/microtubule inhibitor	Relapsed/refractory HL and sALCL	Aug 2011	SGN-35-003	Younes et al. [83]
Gemtuzumab ozogamicin (Mylotarg)	CD33	Cleavable (acid-labile)	Calicheamicin/DNA cleavage agent	CD33 + acute myeloid leukemia (AML)	May 2000 (reapproved 2017)	ALFA-0701	Castaigne et al. [84]
Belantamab mafodotin (Blenrep)	BCMA	Noncleavable	MMAF/microtubule inhibitor	Relapsed/refractory multiple myeloma (MM)	Aug 2020	DREAMM-2	Lonial et al. [85]

Abbreviations: *ALL*, acute lymphoblastic leukemia; *AML*, acute myeloid leukemia; *BCMA*, B-cell maturation antigen; *BR*, bendamustine plus rituximab; *CD19*, cluster of differentiation 19; *CD22*, cluster of differentiation 22; *CD30*, cluster of differentiation 30; *CD33*, cluster of differentiation 33; *CD79b*, cluster of differentiation 79b; *DLBCL*, diffuse large B-cell lymphoma; *DM1*, demethylated maytansine 1; *DM4*, demethylated maytansine 4; *DNA*, deoxyribonucleic acid; *DXd*, deruxtecan; *FRα*, folate receptor alpha; *HCL*, hairy cell leukemia; *HER2*, human epidermal growth factor receptor 2; *HL*, Hodgkin lymphoma; *HR +*, hormone receptor-positive; *MM*, multiple myeloma; *MMAE*, monomethyl auristatin E; *MMAF*, monomethyl auristatin F; *NSCLC*, non-small-cell lung cancer; *PBD*, pyrido benzodiazepine; *PE38*, Pseudomonas exotoxin A fragment; *TF*, tissue factor; *TOP1*, topoisomerase I; *TROP2*, trophoblast cell surface antigen 2; *c-Met*, mesenchymalepithelial transition factor; *mTNBC*, metastatic triple-negative breast cancer; *sALCL*, systemic anaplastic large cell lymphoma

Trastuzumab emtansine is an ADC targeting HER2-positive breast cancer. It utilizes a stable, noncleavable N-succinimidyl-4-(maleimidomethyl) cyclohexane-1-carboxylate (SMCC) linker to conjugate the mAb trastuzumab with the cytotoxic maytansinoid DM1. This design ensures systemic stability and limits the bystander effect, as the resulting Lys-MCC-DM1 metabolite is membrane-impermeable. The Phase III EMILIA trial established trastuzumab emtansine as a second-line standard of care for mBC after it significantly improved progression-free survival (PFS) and overall survival (OS) compared to lapatinib plus capecitabine [24]. Despite its targeted nature, trastuzumab emtansine is associated with low-grade systemic toxicities, including thrombocytopenia and elevated transaminases (please add other common toxicities from T-DM1). Current evidence indicates that these adverse events primarily arise from target-independent uptake of ADCs by nonmalignant cells. Specifically, Fcγ receptor-mediated endocytosis by megakaryocytes and nonspecific pinocytosis by hepatocytes lead to the intracellular degradation of the ADC and subsequent release of the highly potent cytotoxic DM1 payload [25]. We conducted a retrospective analysis and developed a toxicity sum score (TSS) based on platelet counts and liver enzymes toxicities [3]. Our findings revealed that a higher TSS significantly correlated with longer PFS (adjusted HR = 0.67, *P* = 0.02). This was the first report to correlate the systemic toxicities of ADCs such as trastuzumab emtansine with clinical outcome. Further, this suggests that systemic toxicities of ADCs may serve as a predictive biomarker, particularly if noncleavable linkers are used.

Trastuzumab deruxtecan is a newer ADC developed for the treatment of HER2-positive mBC. It contains the same HER2-binding trastuzumab, a cleavable tetrapeptide linker and a potent topoisomerase I inhibitor derivative deruxtecan. DESTINY-BREAST01 trial demonstrated its significant efficacy in refractory HER2-positive mBC [26]. In the intention-to-treat analysis, a response to therapy was reported in 112 patients (60.9%; 95% confidence interval [CI], 53.4 to 68.0). The median duration of follow-up was 11.1 months (range, 0.7–19.9 months). The median response duration was 14.8 months (95% CI, 13.8–16.9 months), and the median duration of PFS was 16.4 months (95% CI, 12.7–not reached months). However, significant systemic toxicities were observed. Grade 3 or higher toxicities included decreased neutrophil count in 20.7% of the patients, anemia in 8.7%, and nausea in 7.6%. On independent adjudication, the trial drug was associated with interstitial lung disease (ILD) in 13.6% of the patients (grade 1 or 2, 10.9%; grade 3 or 4, 0.5%; and grade 5, 2.2%). DESTINY-BREAST01 impressed us not only with its efficacy but its significant systemic toxicities, including the grade 5 ILD.

When we compared the systemic toxicities of trastuzumab deruxtecan with deruxtecan prodrug–related topoisomerase I inhibitors such as topotecan (which is directly active and does not require metabolic activation) and irinotecan (a prodrug whose active metabolite is SN-38) [23, 27, 28], trastuzumab deruxtecan seemed to have even more systemic toxicities than those of the parent drugs’ unconjugated agents (Table 2), despite the shielding of payload toxicities by linking the deruxtecan to trastuzumab. While both the deruxtecan payload and SN-38 function as topoisomerase I inhibitors, they are chemically distinct entities with unique pharmacological profiles. We then compared the systemic toxicities between trastuzumab deruxtecan and trastuzumab emtansine from the DESTINY-BREAST03 phase 3 trial to compare the efficacy and safety of trastuzumab deruxtecan with those of trastuzumab emtansine in patients with HER2-positive mBC previously treated with trastuzumab and a taxane. Both ADCs have the same HER2-targeting trastuzumab but different linker designs (noncleavable vs. cleavable) and payloads (emtansine vs. topoisomerase I inhibitor derivative). Trastuzumab deruxtecan indeed seemed to have more severe systemic toxicities as previously discussed with a significantly higher occurrence of any grade of adverse event (AE) compared to that of trastuzumab emtansine, except for thrombocytopenia [29].

**Table 2:** Comparison of systemic toxicities among topoisomerase inhibitors and trastuzumab deruxtecan

Adverse Event	Irinotecan		Topotecan		Trastuzumab deruxtecan	
**Number of patients (%)**	**Any grade**	**Grade ≥ 3**	**Any grade**	**Grade ≥ 3**	**Any grade**	**Grade ≥ 3**
Any adverse event	-	-	-	-	183 (99.5)	96 (52.2)
Nausea	26–69 (1–95)	26 (14)	9 (45)	0 (0)	143 (77.7)	14 (7.6)
Fatigue	50–60 (2–7)	-	12 (60)	3 (15)	91 (49.5)	11 (6.0)
Alopecia	-	-	2 (10)	0 (0)	89 (48.4)	1 (0.5)
Vomiting	-	26 (14)	5 (25)	0 (0)	84 (45.7)	8 (4.3)
Constipation	26–58 (2–33)	19 (10)	-	-	66 (35.9)	1 (0.5)
Neutropenia	-	42 (22)	11 (55)	19 (48)	64 (34.8)	38 (20.7)
Decreased appetite	36–60 (2–49)	-	-	-	57 (31.0)	3 (1.6)
Anemia	-	13 (7)	14 (70)	9 (45)	55 (29.9)	16 (8.7)
Diarrhea	31–86 (2–33)	42 (22)	6 (30)	0 (0)	54 (29.3)	5 (2.7)
Leukopenia	-	-	-	-	39 (21.2)	12 (6.5)
Thrombocytopenia	-	2 (1)	16 (80)	11 (55)	39 (21.2)	8 (4.3)
Headache	-	-	-	-	36 (19.6)	0 (0)
Cough	-	-	-	-	35 (19.0)	0 (0)
Abdominal pain	-	26 (14)	-	-	31 (16.8)	2 (1.1)
Lymphocytopenia	-	-	11 (55)	8 (40)	26 (14.1)	12 (6.5)
Adverse events of special interest						
Interstitial lung disease	-	-	-	-	25 (13.6)	1 (0.5)
Prolonged QT interval	-	-	-	-	9 (4.9)	-
Infusion-related reaction	-	-	-	-	4 (2.2)	-
Decreased left ventricular ejection fraction	-	-	-	-	3 (1.6)	-

Data are presented as number of patients (percentage). Adverse events graded according to Common Terminology Criteria for Adverse Events (CTCAE) version 5.0. Irinotecan and Topotecan data represent ranges from literature review. Trastuzumab deruxtecan data from DESTINY-Breast01 trial (*N* = 184)

Abbreviations: *CTCAE*, common terminology criteria for adverse events; *LVEF*, left ventricular ejection fraction

It is important to acknowledge the methodological limitations of this indirect, observational comparison. Direct dosing equivalence between an ADC and an unconjugated payload cannot be perfectly established due to fundamentally different pharmacokinetic (PK) profiles and the amount or concentration of active metabolites generated from the prodrugs. Free cytotoxic agents typically exhibit rapid systemic clearance and wide volumes of distribution. In contrast, the monoclonal antibody backbone of an ADC significantly extends the half-life and restricts the initial volume of distribution to the vascular compartment, leading to prolonged, low-dose systemic exposure of the prematurely cleaved payload. Consequently, the observed increases in specific toxicities (such as neutropenia or ILD) may reflect these altered exposure dynamics and the potent bystander efficacy of the drug, rather than just the absolute dose administered. Future clinical studies incorporating robust pharmacokinetic and pharmacodynamic (PK/PD) modeling are essential to definitively quantify the relationship between free payload circulation, therapeutic efficacy, and systemic adverse events.

## ADC linker designs and systemic toxicities

A central enigma in modern oncology is why the conjugation of deruxtecan to trastuzumab (as seen in trastuzumab deruxtecan) fails to provide a sufficient pharmacological shield to prevent the cytotoxic payload from inducing significant systemic toxicities. Given that both trastuzumab emtansine and trastuzumab deruxtecan utilize the identical monoclonal antibody backbone targeting the HER2 receptor, the divergent safety profiles and systemic adverse events observed in clinical practice must be attributed to the architectural differences in their linker–payload systems.

A primary distinction lies in the biochemical nature of the linkage: trastuzumab emtansine employs a robust, noncleavable thioether linker (MCC), whereas trastuzumab deruxtecan utilizes a cathepsin-cleavable tetrapeptide-based linker. We hypothesize that the off-target systemic toxicities associated with these ADCs are fundamentally dictated by this linker design. ADCs incorporating noncleavable linkers generally exhibit a superior systemic safety profile because the liberation of the payload is strictly sequestered within the lysosomal compartment of cells expressing the specific biomarker. In this model, the payload remains covalently bound to the antibody throughout circulation, preventing premature release.

Conversely, ADCs utilizing cleavable linkers, while highly effective at inducing a bystander effect, are inherently more prone to “leakiness” or premature cleavage within the systemic circulation or the extracellular space before reaching the intended malignant tissue. This premature release of the free, membrane-permeable payload leads to unintended exposure in healthy tissues, thereby narrowing the therapeutic index and increasing the incidence of systemic toxicity. Consequently, the trade-off for the enhanced potency offered by cleavable technology appears to be a heightened risk of off-target effects that are absent in more stable, noncleavable configurations. We hypothesized that the systemic toxicities from ADCs were related to their linker design. ADCs with noncleavable linkers possess less systemic toxicities since the payload is released only in tumor cells bearing the respective biomarkers while those with cleavable linkers cause more systemic toxicities due to the premature payload release before they reach the target cells.

We conducted a meta-analysis, in 2022, of the AEs caused by all FDA-approved ADCs from 11 Phase II/III clinical trials involving 2417 patients [30]. We found for the first time that ADCs with cleavable linkers had a significantly higher rate of grade ≥ 3 AEs (47%) compared to those with noncleavable linkers (34%), with a weighted risk difference of −12.9%. Specifically, cleavable linkers were associated with significantly higher rates of severe neutropenia (−9.1% risk difference) and anemia (−1.7%). In addition, we demonstrated that higher levels of free payload were released into circulation from ADCs with cleavable linkers compared to those with noncleavable linkers. We subsequently performed a systemic review and meta-analysis of forty clinical trials on 11 FDA-approved ADCs involving 7879 patients, including 9 ADCs with cleavable linkers (*N* = 2985) and 2 with noncleavable linkers (*N* = 4894) [11]. Our studies supported our hypothesis that ADCs with cleavable linkers result in premature payload release, leading to increased systemic free payload concentrations and more frequent and server systemic toxicities. Our observation was subsequently supported by other investigators and publications [7, 9, 31].

Beyond the intrinsic chemical stability of the linker, the systemic safety profile of ADCs with cleavable linkers is also influenced by the complex biological environment. For instance, circulating HER2-positive exosomes and the shed extracellular domain (ECD) of the HER2 receptor—frequently observed in advanced breast cancer—can act as “decoy” targets[32]. These non-cell-bound entities may intercept ADCs like trastuzumab deruxtecan in the circulation or tumor interstitial, where they can render the ADCs unable to target tumor cells and the antigen–antibody-bound ADCs opsonized and internalized by macrophages/phagocytes via Fc receptors. This mechanism likely contributes to the unintended systemic exposure of potent, membrane-permeable payloads, thereby broadening the spectrum of off-target toxicities in patients with high target antigen shedding.

## Efficacy and toxicity trade off by ADC linker design

The therapeutic efficacy and pharmacological profile of ADCs are governed by a complex interplay of structural parameters, including antibody affinity, linker cleavability, payload potency, and the drug-to-antibody ratio (DAR). A critical design choice in this architecture is the selection between cleavable and noncleavable linkers, a decision that fundamentally dictates the equilibrium between antitumor potency and systemic toxicity. Cleavable linkers are engineered to liberate their cytotoxic cargo in response to specific environmental triggers, such as acidic pH or proteolytic enzymes, which can occur both within the systemic circulation and the tumor microenvironment. This mechanism facilitates biomarker-independent killing via a potent bystander effect, particularly when paired with a high DAR (e.g., 8:1) and membrane-permeable payloads. However, the inherent risk of this design is the premature release of the payload before reaching target cells, which inevitably increases off-target systemic toxicities.

**Fig. 1:**
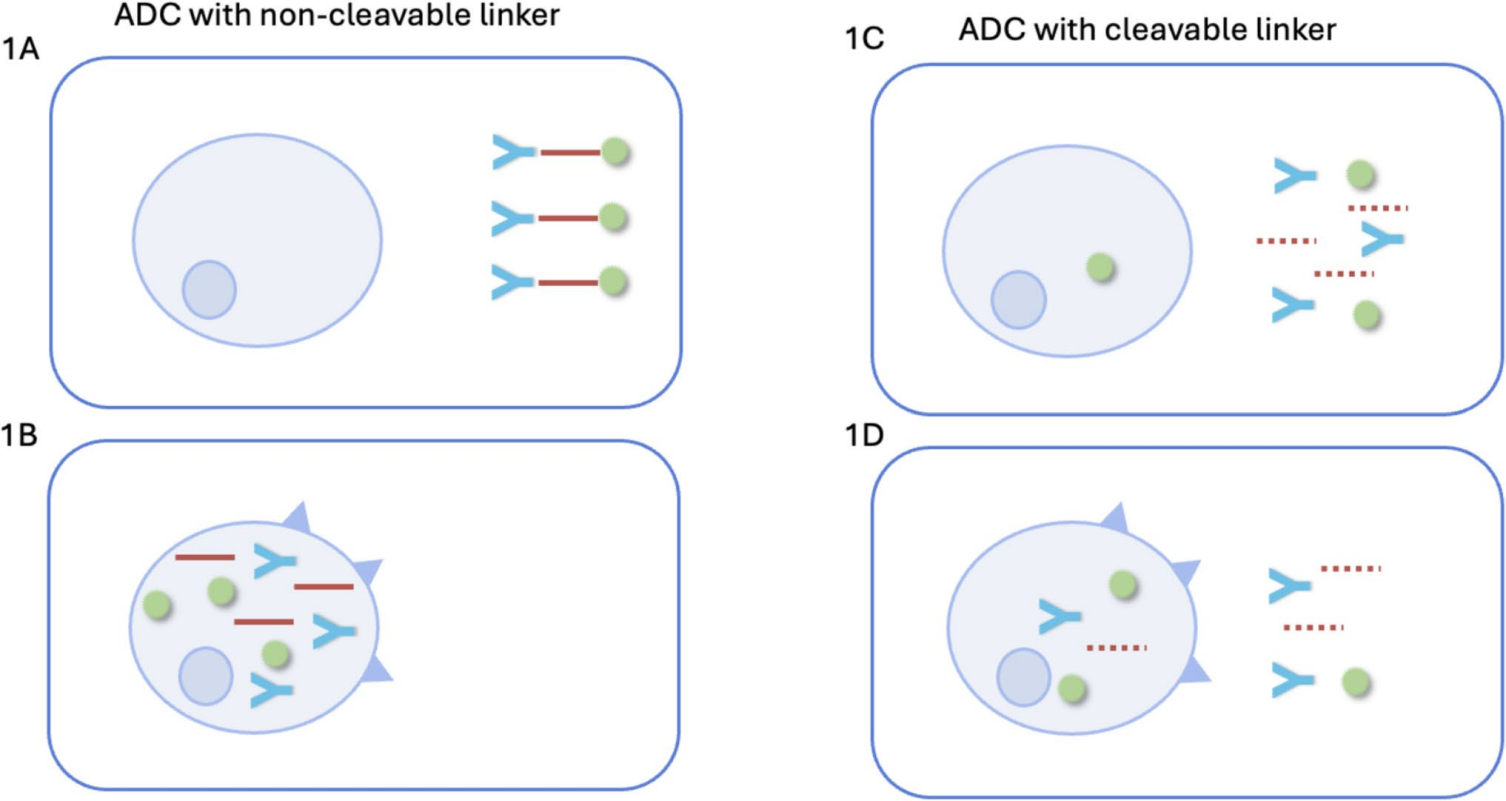
Antibody–drug conjugate (ADC) linker mechanisms and bystander effect. **A**, **B** Noncleavable linkers release impermeable payloads only in antigen-positive cells after degradation. **C**, **D** Cleavable linkers release permeable payloads upon cleavage, enabling bystander killing of antigen-negative cells. ADC, antibody–drug conjugate

In contrast, noncleavable linkers require the complete lysosomal degradation of the antibody backbone to release the active drug, strictly confining cytotoxic activity to cells that express the target biomarker. While this design limits bystander destruction, it generally offers a more targeted safety profile. Indeed, research into trastuzumab emtansine suggests that its associated toxicities correlate closely with tumor lysis, implying that systemic exposure primarily originates from destroyed tumor cells rather than from premature linker cleavage in the blood [8]. These fundamental mechanistic differences are illustrated in Fig. 1, where ADCs with noncleavable linkers (Fig. 1B) require internalization by antigen-expressing cells for payload release, whereas those with cleavable linkers (Fig. 1C and D) can mediate payload release extracellularly, thereby enabling the bystander effect.

The clinical significance of these design choices was highlighted in the DESTINY-Breast01 trial, which demonstrated the superior efficacy of trastuzumab deruxtecan over trastuzumab emtansine. This success is likely attributable to its higher DAR, cleavable linker, and topoisomerase I inhibitor payload, which effectively overcomes tubulin-based resistance. However, achieving a high therapeutic index remains a delicate balancing act. Currently, most FDA-approved ADCs utilize cleavable linkers and carry a burden of considerable systemic toxicity, whereas the few approved noncleavable designs show more limited, albeit distinct, toxicity profiles. Interestingly, despite the perceived stability of noncleavable linkers, recent analyses reveal they are associated with unique risks, such as a 14.29-fold increase in ophthalmologic events and specific pulmonary or hematologic toxicities [33]. Ultimately, ADC development must continue to refine these mechanisms to optimize the therapeutic index, balancing the superior efficacy of cleavable technologies against the specialized safety profiles of noncleavable designs.

The structural complexity of ADCs extends beyond linker chemistry to include antibody isotypes and payload characteristics. Most current ADCs utilize an IgG1 backbone, which, while promoting potent tumor targeting and antibody-dependent cellular cytotoxicity (ADCC), possesses high affinity for Fcγ receptors (FcγRs) on nonmalignant cells, such as megakaryocytes and hepatocytes [34]. This interaction can drive target-independent uptake and subsequent toxicity, a factor that must be considered alongside linker cleavability. Regarding payloads, microtubule inhibitors (e.g., DM1, DM4, and MMAE) and DNA-damaging topoisomerase I inhibitors (e.g., DXd and SN-38) exhibit distinct pharmacological behaviors [35]. While microtubule inhibitors primarily target mitotically active cells, DNA-damaging agents can induce apoptosis regardless of the cell cycle phase. The class of payloads also determines the toxicities of ADCs, including unique toxicities associated with each class of payload, such as neuropathy associated with the tubulin-targeting payloads and interstitial pneumonitis associated with the DNA-damaging payloads. The synergy between the antibody isotype, payload potency, and linker chemistry collectively determines the equilibrium between clinical efficacy and systemic safety.

## ADC biomarker dependence or independence

While ADCs featuring noncleavable linkers typically operate through a strictly biomarker-dependent mechanism, a growing body of evidence suggests that many ADCs utilizing cleavable linkers exhibit unexpected clinical efficacy even in biomarker-negative tumors. This phenomenon challenges the traditional “magic bullet” theory, which posits that ADCs function solely as targeted delivery vehicles. Previous research has demonstrated that ADCs with cleavable linkers result in significantly higher levels of free plasma payload compared to their noncleavable counterparts. This observation led to the hypothesis that liberated payloads—whether released in the systemic circulation or within the tumor interstitial space—may facilitate tumor cell destruction in a biomarker-independent manner, effectively bypassing the requirement for high-density target antigen expression [11]. The pharmacological behavior of these “leaky” linkers suggests that the therapeutic effect may be driven as much by local chemotherapy-like diffusion as by site-specific antibody binding.

This initial speculation has gained substantial clinical support from the DAISY trial, which evaluated the efficacy of trastuzumab deruxtecan in metastatic breast cancer (mBC) across varying degrees of HER2 expression. The trial reported confirmed overall response rates (ORR) of 70.6% in the HER2-overexpressing cohort (*N* = 74) and 37.5% in the HER2-low cohort (*N* = 74). Most notably, trastuzumab deruxtecan achieved a 29.7% ORR in the HER2-zero (IHC 0) cohort (*N* = 40) [36], a population traditionally considered entirely nonresponsive to HER2-targeted therapies. This suggests that the potent DXd payload, once cleaved in the circulation and preferentially in the tumor microenvironment (TME), can target and eradicate cells regardless of their HER2 status. To explain why this payload release appears to occur preferentially in TME rather than systemically, it is critical to consider the unique biochemical conditions of the TME. The localized extracellular space within tumors is characterized by an acidic pH and high concentrations of active proteases, such as Cathepsin L, which actively and preferentially cleave the linker. In contrast, this cleavable linker remains relatively stable in the neutral, protease-poor environment of the systemic circulation, thus concentrating the payload release and subsequent bystander effect within the tumor bed [37]. Similar clinical activity was observed in the DESTINY-Lung01 trial, where trastuzumab deruxtecan demonstrated efficacy in advanced non-small-cell lung cancer (NSCLC) across diverse HER2 expression levels, including both IHC 3 + and 2 + patients [38]. Furthermore, trastuzumab deruxtecan yielded a 55% objective response rate in patients with metastatic HER2-mutant NSCLC, further underscoring its versatility and its ability to function effectively even when the absolute quantity of surface antigen is limited.

Taken together, these findings fundamentally challenge the conventional ADC paradigm, which dictates that therapeutic efficacy must strictly correlate with target antigen density. The traditional reliance on IHC scoring as a binary “go/no-go” signal for treatment is increasingly viewed as insufficient for next-generation ADCs. While the FDA has approved several ADCs alongside companion diagnostic (CDx) tests—such as trastuzumab emtansine for HER2-positive cases, mirvetuximab soravtansine-gynx for FRα-positive tumors, and others targeting BCMA, CD30, or CD33—a significant number of ADCs have been granted approval without any specific biomarker requirement, as detailed in Table 3. This regulatory shift reflects a growing recognition that the bystander effect and extracellular payload release can provide meaningful clinical benefits. Consequently, there is an ongoing and vigorous debate within the oncology community regarding whether biomarker expression is a prerequisite for ADC-mediated tumor destruction or if the systemic and microenvironmental release of potent payloads can independently drive clinical benefit. As we move toward a more nuanced understanding of ADC pharmacology, the industry may need to redefine “targetability” to account for the complex interplay between linker stability, payload permeability, and the heterogeneous nature of the tumor microenvironment.

**Table 3:** Biomarkers and companion diagnostics modalities for FDA-approved antibody–drug conjugates (ADCs)

ADC (trade name)	Target biomarker (CDx)	Relevant indication(s) (abbreviated)	Testing method(s)/diagnostic criteria	Source
Trastuzumab deruxtecan (Enhertu)	HER2 (protein expression/gene status)	HER2-positive/HER2-low metastatic breast cancer; HER2 + gastric/GEJ cancer; HER2-mutant NSCLC	IHC (IHC 3 + or IHC 1 +/2 + and FISH-negative for HER2-low)/FISH/NGS (for HER2 mutations)	Tissue/plasma
Trastuzumab emtansine (Kadcyla)	HER2 (protein expression/gene status)	HER2-positive metastatic breast cancer (mBC) and adjuvant	IHC (3 +) and/or FISH (gene amplification)	Tissue
Mirvetuximab soravtansine (Elahere)	Folate receptor α (FRα)	FRα-high platinum-resistant ovarian cancer	IHC (high expression required; ≥ 75% of cells staining with 2 + intensity)	Tissue
Telisotuzumab vedotin-tllv	c-Met	locally advanced or metastatic NSCLC with high c-Met protein overexpression	IHC (high expression required; ≥ 50% of cells staining with 3 + intensity)	Tissue
Belantamab mafodotin (Blenrep)	B-cell maturation antigen (BCMA)	Relapsed/refractory multiple myeloma (MM)	IHC (confirmation of BCMA expression)	Tissue/bone marrow
Brentuximab vedotin (Adcetris)	CD30	Hodgkin lymphoma (HL); systemic anaplastic large-cell lymphoma (sALCL); CTCL	IHC (confirmation of CD30 expression)	Tissue
Gemtuzumab ozogamicin (Mylotarg)	CD33	CD33-positive acute myeloid leukemia (AML)	Flow cytometry (confirmation of CD33 expression)	Blood/bone marrow

Abbreviations: *ADC*, antibody–drug conjugate; *CDx*, companion diagnostic; *HER2*, human epidermal growth factor receptor 2; *NSCLC*, non-small-cell lung cancer; *FRα*, folate receptor alpha; *TROP2*, trophoblast cell surface antigen 2; *TF*, tissue factor; *BCMA*, B-cell maturation antigen; *HL*, Hodgkin lymphoma; *DLBCL*, diffuse large B-cell lymphoma; *ALL*, acute lymphoblastic leukemia; *AML*, acute myeloid leukemia; *MM*, multiple myeloma; *sALCL*, systemic anaplastic large-cell lymphoma; *CTCL*, cutaneous T-cell lymphoma

Sacituzumab govitecan is an ADC with a cleavable linker targeting the tumor-associated calcium signal transducer-2 (TROP-2) protein, highly expressed in cancers such as breast, bladder, and lung. It is effective in mTNBC and mUC, with ORR varying by cancer type and population. In the ASCENT trial, it improved ORR vs. chemotherapy in pretreated mTNBC [39]. In TROPHY-U-01, ORR was 27% in mUC post platinum and checkpoint inhibitors [40]. Activity is also seen in EC and SCLC, though ≥ 3 TRAEs occurred in 73% of EC patients [41, 42]. Approval for mTNBC/mUC does not require TROP-2 testing. Analyses show a loose positive correlation between TROP-2 expression (*H*-score) and outcomes (PFS and OS), yet activity remains even with low expression [43].

Sacituzumab govitecan employs a uniquely engineered linker–payload architecture. It utilizes a proprietary hydrolyzable CL2A linker that is moderately stable and pH-sensitive, conjugating the antibody to the payload SN-38. Although SN-38 acts as a topoisomerase I inhibitor similarly to the deruxtecan (DXd) payload used in other ADCs, the two are chemically distinct entities. Because SN-38 possesses moderate intrinsic cytotoxicity compared to highly potent exatecan derivatives, sacituzumab govitecan can be engineered with a remarkably high drug-to-antibody ratio (DAR) of approximately 7.6 without compromising systemic pharmacokinetic stability or inducing prohibitive aggregation [44].

This high DAR, integrated with the specific hydrolyzable properties of the CL2A linker, enables robust dual mechanisms of action: classical biomarker–dependent intracellular targeting following lysosomal degradation, and a potent biomarker-independent bystander effect. Crucially, the pH-sensitive nature of the CL2A linker permits the hydrolysis and premature release of free, membrane-permeable SN-38 directly into the acidic tumor microenvironment (TME) prior to cellular internalization [45]. This dynamic extracellular payload release ensures that adjacent TROP-2-negative or TROP-2-low tumor cells are eradicated. As a result, sacituzumab govitecan demonstrated its significant activities in TROP-2-positive TNBC tumor cells and moderate activities in TROP-2-negative cells. [46].

Mirvetuximab soravtansine is an ADC targeting folate receptor alpha, with a tubulin-targeting DM4 payload and a sulfo-SPDB (sulfosuccinimidyl 4-(N-maleimidomethyl) cyclohexane-1-carboxy-(3-sulfo-4-nitrophenyl)−1,3-dihydro-2H-benzimidazole-2-one) cleavable disulfide-containing linker. It has significant activity in platinum-resistant ovarian cancer [47].

To fully contextualize its clinical efficacy, it is essential to examine its specific linker-payload synergy. The sulfo-SPDB linker features a sterically hindered disulfide bond designed to maintain systemic stability, yet it is efficiently cleaved upon target-mediated internalization within the reductive, glutathione-rich intracellular environment of the tumor cell [48]. This intracellular cleavage liberates the potent maytansinoid DM4. Crucially, unlike the membrane-impermeable metabolites typically generated by noncleavable linkers, free DM4 is highly lipophilic and uncharged. This allows it to readily diffuse out of the targeted cell and into the adjacent tumor microenvironment, exerting a profound cytotoxic effect on neighboring cells regardless of their FRα expression status[49].

The FDA’s initial accelerated approval of the cleavable-linker ADC mirvetuximab soravtansine mandated a CDx with a high FRα positivity threshold (≥ 75% of tumor cells). However, the subsequent lowering of this threshold to ≥ 50% for the full approval, backed by clinical efficacy, critically illustrates that for ADCs with a cleavable linker and a bystander effect, the required level of target biomarker expression for patient selection can be fluid [50]. This suggests that the potent bystander killing effect, driven by DM4’s robust membrane permeability, can compensate for lower target homogeneity and patchy antigen distribution [51]. Prematurely released payload of mirvetuximab soravtansine in the TME also targets the tumor cells with less or no FRα expression. By effectively neutralizing FRα-negative or low-expressing clones within a heterogeneous tumor, this dynamic mechanism potentially expands the treatable population beyond what was initially defined by a strict CDx cutoff.

Recent research challenges the traditional understanding of ADC mechanisms. Tsao et al. demonstrated that the efficacy of trastuzumab deruxtecan in HER2-low or HER2-negative breast cancer does not strictly require receptor binding or internalization [12]. Instead, activity is mediated by extracellular proteases cathepsin L (CTSL) in the tumor microenvironment, which release the payload prematurely. Similarly, Major et al. found that HER2 surface expression alone poorly predicts cytotoxicity [52]. The critical determinant is reaching a minimum threshold of payload delivery to lysosomes, rather than total surface levels. These findings suggest current HER2 testing may unnecessarily restrict patient access to therapy. To further investigate, a Phase II trial (NCT pending) is evaluating trastuzumab deruxtecan in 50 patients with HER2-zero (IHC 0) mBC [53]. By utilizing serial biomarker and ctDNA analysis, the study aims to confirm that ADCs with cleavable linkers can release payloads extracellularly, killing tumor cells independently of biomarker presence. This shifts the paradigm from biomarker-dependent targeting to microenvironment-mediated payload release.

These observations further reinforce our initial hypothesis that ADC’s antitumor activity may operate through mechanisms beyond tumor antigen-dependent internalization [11]. The current evidence suggests ADC efficacy transcends simple antigen-dependent internalization. ADCs with noncleavable linkers primarily rely on target-dependent internalization, resulting in biomarker-dependent activity and lower systemic toxicity. Conversely, those with cleavable linkers can act in a biomarker-independent manner due to premature payload release in systemic circulation or the tumor microenvironment (TME). While the “bystander effect” was traditionally attributed to the membrane diffusion of intracellularly released payloads, recent studies by Tsao et al. suggest it may result exclusively from extracellular linker cleavage within the TME. This challenges the relevance of payload membrane permeability, as little to no intracellularly released payload appears to contribute to the bystander effect. Taken together, current literature suggests that ADCs with cleavable linkers utilize three pathways: (1) target-dependent internalization, (2) target-independent systemic release, and (3) target-independent TME release. This allows them to bypass intratumoral heterogeneity and biomarker limitations, unlike noncleavable ADCs which remain strictly biomarker dependent. 

## ADCs with the next-generation linker designs

**Fig. 2:**
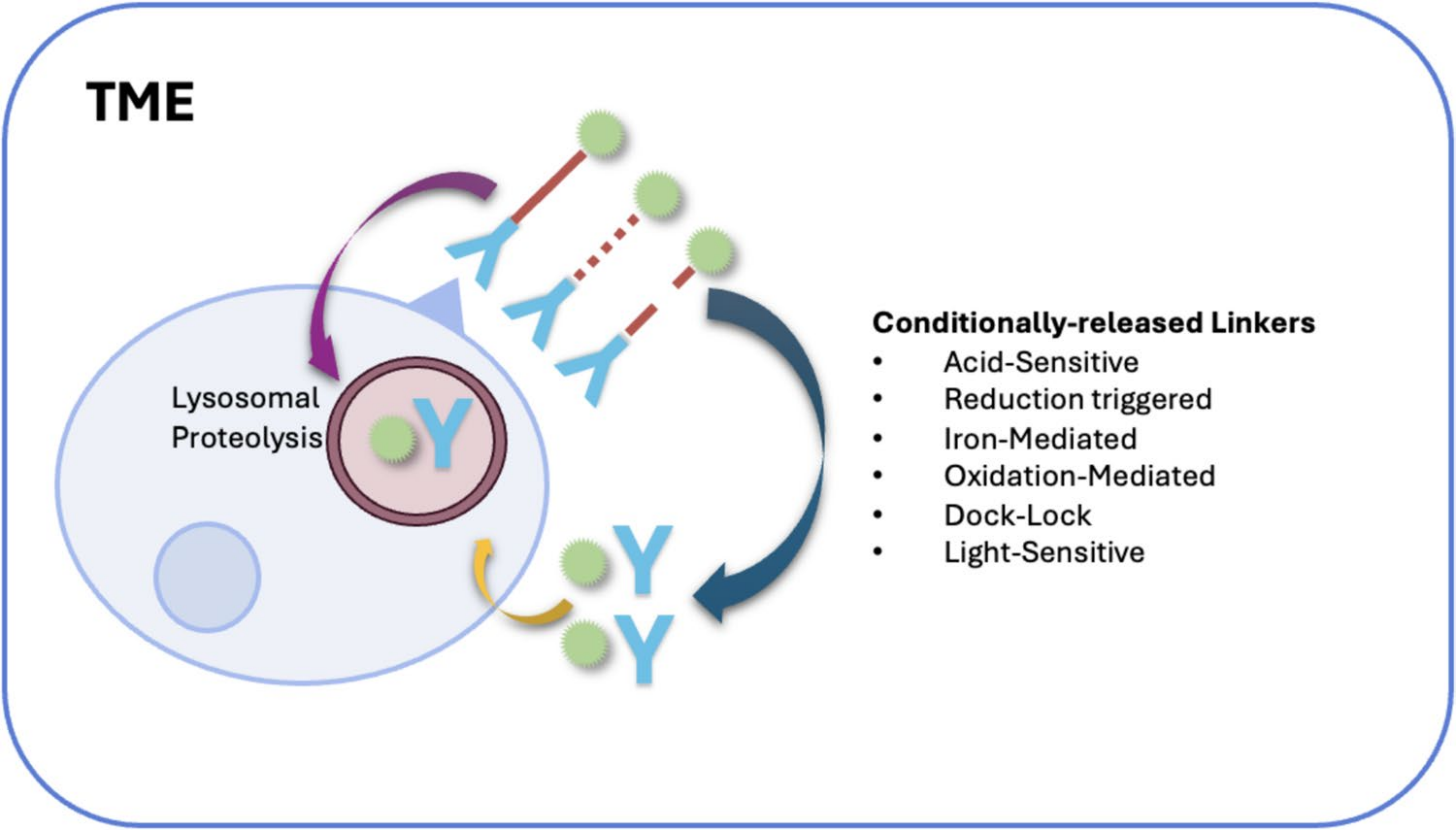
Next-generation conditionally cleavable linkers for TME-specific payload release. Noncleavable linkers (magenta arrow) require antigen-mediated internalization and subsequent trafficking to the lysosome (depicted as a distinct intracellular compartment) for payload release. Advanced cleavable linkers (dark blue arrow) are engineered for selective cleavage by TME stimuli (e.g., pH, proteases, and redox), enabling biomarker-independent killing via bystander effect. TME, tumor microenvironment

It is now evident that ADCs with noncleavable linkers function in a biomarker-dependent manner and only release the payload after they enter the target tumor cells with reduced efficacy and limited systemic toxicities while those with cleavable linkers act in both biomarker-dependent and independent manners and readily release the payload before they reach the tumor cells, with increased efficacy and toxicities. To balance the trade-off between the superior efficacy of cleavable linkers and their associated increased systemic toxicity compared to noncleavable platforms [54]. Next-generation linker strategies aimed at achieving tumor-selective activation are depicted in Fig. 2. These “conditionally released” linkers are designed to be triggered specifically by the unique tumor microenvironment (e.g., low pH and specific enzymes), thereby maximizing antitumor efficacy while minimizing off-target toxicity [55–57].

### Lysosome-dependent linkers

The engineering of lysosome-dependent linkers represents a sophisticated approach to enhancing the therapeutic index of ADCs by ensuring that the cytotoxic payload is liberated only within the acidic, enzyme-rich environment of the target cell. These linkers are specifically designed for exceptional systemic stability, remaining largely inert while in circulation to prevent premature drug release. Their activation is strictly contingent upon antigen-mediated internalization and subsequent trafficking to the lysosomal compartment, a mechanism that guarantees a high degree of target specificity and minimizes off-target toxicity.

Among the most widely utilized are protease-sensitive linkers, particularly the dipeptide motifs valine-citrulline (Val-Cit) and valine-alanine (Val-Ala). These sequences are engineered to be recognized and cleaved by lysosomal proteases, most notably cathepsin B. Because these proteases are sequestered within the cell and their precursors are inactive in the bloodstream, these linkers maintain a robust plasma profile while ensuring rapid payload release upon internalization, establishing them as a foundational cornerstone of modern ADC technology [58, 59].

Further expanding this toolkit are β-glucuronide linkers, which are highly hydrophilic and serve as substrates for β-glucuronidase, an enzyme concentrated within the lysosomal lumen [60, 61]. Beyond their precise cleavage mechanism, these linkers offer a significant biochemical advantage by dramatically increasing the solubility of often-hydrophobic cytotoxic payloads. This increased hydrophilicity reduces the risk of ADC aggregation during manufacturing and storage, while simultaneously improving the overall pharmacokinetic profile *in vivo*.

Finally, arylsulfate-based linkers represent a more recent innovation in the pursuit of absolute intracellular precision. These linkers act as specific substrates for sulfatase enzymes, which exhibit high intracellular catalytic activity but remain virtually inactive in the extracellular space [62]. This sharp disparity in enzymatic activity ensures that the ADC remains a stable prodrug throughout its journey in the systemic circulation, with activation occurring exclusively after successful internalization into the target cell’s lysosomal machinery.

### Conditionally released linkers

This category encompasses linkers designed to respond to specific physicochemical conditions of the TME or external stimuli, enabling payload release prior to or independent of cellular internalization. This can facilitate a potent bystander effect but requires exquisite selectivity to minimize off-target toxicity. Acid-sensitive linkers, for example hydrazone-based types, hydrolyze in mildly acidic environments. While they can be triggered by the slightly acidic tumor microenvironment, which typically has a pH around 6.5 to 7.0, their most robust cleavage occurs in the more acidic endosomal and lysosomal compartments. Endosomes have a pH of approximately 5.5 to 6.0, and lysosomes are more acidic at about pH 4.5 to 5.0. These linkers represent a hybrid mechanism but are often categorized in this group because of their responsiveness to the TME [63, 64]. Reducible linkers, such as those containing a disulfide bond, exploit the steep gradient in reducing potential between the oxidizing extracellular space and the reducing intracellular cytoplasm, which has a high glutathione concentration. They are cleaved upon cellular entry, often within the endosomal compartment before the payload reaches the lysosome [65–67]. Dual-mechanism or “double-lock” linkers are advanced systems that require two independent triggers for activation. An example is a linker combining a β-glucuronide moiety with an acid-sensitive trigger. This design provides an additional layer of specificity to prevent premature payload release in healthy tissues [54, 67–69]. Fe (II)-cleavable linkers utilize the dysregulated iron metabolism in cancer cells. They undergo cleavage via a Fenton reaction in the presence of intracellular ferrous ions [70, 71]. Photo-responsive linkers offer the highest degree of spatiotemporal control. They remain inert until activated by an external light source, such as ultraviolet or near-infrared light, which completely decouples the release mechanism from inherent biological variables [72].

The selection and engineering of linkers in ADCs represent a fundamental trade-off between biomarker-dependent specificity and the broader bystander effect, a balance that directly dictates the drug’s therapeutic index. Noncleavable linkers offer high systemic stability but strictly require biomarker-mediated internalization, as the cytotoxic payload is only released following total lysosomal degradation within the target cell. While this localized release significantly reduces off-target toxicity, it often limits overall efficacy by preventing the drug from reaching neighboring, biomarker-negative cells. In contrast, cleavable linkers are designed to respond to physiological triggers such as pH shifts or enzymatic activity, allowing for both biomarker-dependent and -independent release. This enables the bystander effect, where the payload diffuses to kill surrounding tumor cells, though it carries the inherent risk of premature cleavage in circulation, leading to increased systemic toxicity. To bridge this gap, next-generation ADC development focuses on selective activation within the unique tumor microenvironment—exploiting localized hypoxia, extracellular acidity, and high enzymatic density—to ensure the payload is liberated only at the tumor site, thereby maximizing antitumor potency while maintaining a superior safety profile.

## Conclusion

ADCs represent a transformative class of therapeutics in oncology by enabling targeted, biomarker-directed cytotoxic delivery, thereby sparing normal tissues. All current ADCs, however, cause systemic toxicity. Noncleavable linkers act in a strictly biomarker-dependent manner, correlating with lower toxicity. Cleavable linkers function via both biomarker-dependent and biomarker-independent mechanisms, offering greater potency but increased toxicity. In conclusion, the design of the ADC linker, particularly its cleavability, is a primary determinant of its mechanism of action, balancing biomarker dependence with systemic toxicity. The development of next-generation, conditionally cleavable linkers that are selectively activated within the tumor microenvironment holds the promise of achieving a superior therapeutic index, thereby fully realizing the potential of the “magic bullet.” Research into conditionally released linkers aims to achieve this improved therapeutic index for broader clinical application, among other novel strategies.

## Data Availability

No datasets were generated or analyzed during the current study.

## Declarations

### AI statement

Artificial intelligence (AI) tools were not used in the preparation and revision of this manuscript. All authors take full responsibility for the final integrity, scientific accuracy, and originality of the publication.
